# Threshold Uniformity Improvement in 1b Quanta Image Sensor Readout Circuit

**DOI:** 10.3390/s22072578

**Published:** 2022-03-28

**Authors:** Zhaoyang Yin, Jiaju Ma, Saleh Masoodian, Eric R. Fossum

**Affiliations:** 1 Thayer School of Engineering, Dartmouth College, Hanover, NH 03755, USA; eric.r.fossum@dartmouth.edu; 2 Gigajot Technology, Inc., Pasadena, CA 91107, USA; jiaju.ma@gigajot.tech (J.M.); sm@gigajot.tech (S.M.)

**Keywords:** single-bit quanta image sensor (QIS), 1-bit quantizer, high speed, low noise, readout cluster uniformity

## Abstract

A new readout architecture for single-bit quanta image sensor (QIS) consisting of a capacitive transimpedance amplifier (CTIA) before a 1-bit quantizer to improve the threshold uniformity of the readout cluster is proposed in this paper. The 1-bit quantizer in the previous single-bit QIS had significant threshold non-uniformity likely caused by the fluctuation of the common-mode voltage of the jot output. To guarantee the stability of the common-mode voltage of input signals fed to the 1-bit quantizer, the CTIA is added before the 1-bit quantizer. A pipeline operation mode is also proposed so the CTIA and 1-bit ADC can work at the same time, thereby reducing the CTIA power consumption. A 2048 × 1024 high-speed test chip was implemented with 45 nm/65 nm stacked backside illuminated (BSI) CMOS image sensor (CIS) process and tested. According to the measured D-log-H results, a good threshold uniformity in the range of 0.3 to 0.8 e− for all readout clusters is demonstrated at 500 frame per second (fps) equivalent timing with 68 mW power consumption.

## 1. Introduction

The QIS concept was first introduced in 2005 as a paradigm shift to take advantage of shrinking pixel sizes [1]. The small pixels in a QIS are called jots and have deep sub-diffraction-limit (SDL) pitch, small full-well capacity (FWC) and deep sub-electron read noise (DSERN) (e.g., <0.6 μm, 100 e− and <0.3 e− r.m.s., respectively). Many efforts have been made to improve the jot performance, including increasing conversion gain (CG) and reducing readout noise [2,3,4,5,6].

Besides the jot design, another challenge in realizing the QIS concept in a CMOS image sensor (CIS) process is implementing high-speed, low-noise and low-power readout circuitry. An initial exploration of readout circuits was made by designing a 1-Mpixel single-bit image sensor with a conventional 3T pixel operating at 1000 fps [7]. The charge-transfer amplifiers (CTAs) were first introduced to implement single-bit column-parallel analog-to-digital converters (ADCs) [7]. A 1-Mjot photon-counting QIS using the prior CTA-based 1-bit ADC was designed with new cluster-parallel architecture in a 3D stacked process [8]. The threshold of the 1-bit ADC is set to be 0.5 e− (half of the jot CG) to determine the readout of one or more photoelectrons. However, ADCs in a CIS usually have bit depths greater than 10 bits [9,10]. The 1-bit ADC accomplishes conversion within one clock cycle and does not utilize extra conversion steps to overcome some non-ideal factors such as offset and noise. If the extra steps are applied to the 1-bit ADC, its high-speed advantage is eroded. However, this makes the design of a 1-bit ADC complex and difficult. The previous 1-bit ADC [8] based on CTA readout circuits had significant threshold non-uniformity, likely due to the fluctuation of the common-mode voltage of the jot outputs. A complete redesign of the readout circuit is presented in this work as an attempt to address this challenge.

In this paper, a new architecture for 1-bit readout circuits is proposed. A CTIA is added before a 1-bit quantizer to guarantee the stability of the common-mode voltage of the input signals. To remove the offset caused by the CTIA, a correlated double sampling (CDS) block is utilized to store two signals, one for the reset voltage of the CTIA and the other for the amplified difference between the jot reset signal and exposure signal. The capacitors in the CDS block are large enough to ensure the kT/C noise incurred in the sample and hold process is negligible when compared to the total temporal noise. Although the introduction of the CTIA helps to stabilize the threshold across all of the readout clusters, it results in high power consumption. A pipeline operation mode is also proposed to reduce the power consumption of the CTIA. The CDS block stores two sequential output signals and one is used by the quantizer while the other is being sampled in ping-pong fashion. Thus, the CTIA and 1-bit quantizer work at the same time. The D-log-H characterization is used to evaluate the performance of the readout cluster. The experimental results show the 1-bit readout circuits reach 0.5 e− threshold and a good threshold uniformity across the whole array is demonstrated, providing meaningful guidance for the future design of single-bit, QIS devices.

The rest of this paper is organized as follows. The architecture of the single-bit QIS is presented in Section 2. The detailed designs of different blocks in the sensor—CTIA, CDS, 1-bit quantizer and digital circuitry—are presented in Section 3. Section 4 explains how the uniformity of the readout clusters is improved from the previous design. Section 5 reports the experimental results based on the D-log-H methodology and shows a good threshold uniformity of all readout clusters. Sample images are also presented to verify the performance of the sensor. Finally, Section 6 concludes the paper.

## 2. Sensor Architecture

**Figure 1:**
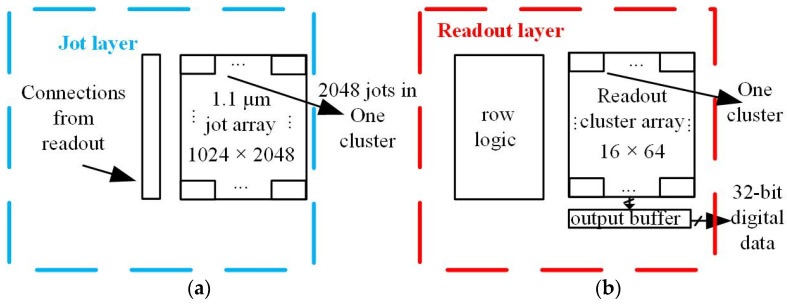
Sensor diagrams: (**a**) diagram of the jot wafer and (**b**) diagram of the readout wafer.

The single-bit QIS was implemented in a TSMC 45 nm/65 nm stacked BSI CIS process, of which the floorplan is shown in Figure 1. The jot layer and readout layer are implemented on two different wafers, connected through high-density hybrid bonding using Cu-Cu bonding pads (“Cu pads”). The jot array is 1024 V × 2048 H jots with 1.1 μm pitch. The jots and readout are designed for cluster-parallel readout with cluster size 16 V × 64 H. One cluster includes 2048 jots. The digital outputs of the readout array are sent to an output buffer array on the bottom of the array and 32 clusters are readout at the same time. The details of the readout circuitry are given in the following sections.

## 3. Readout Circuit

**Figure 2:**
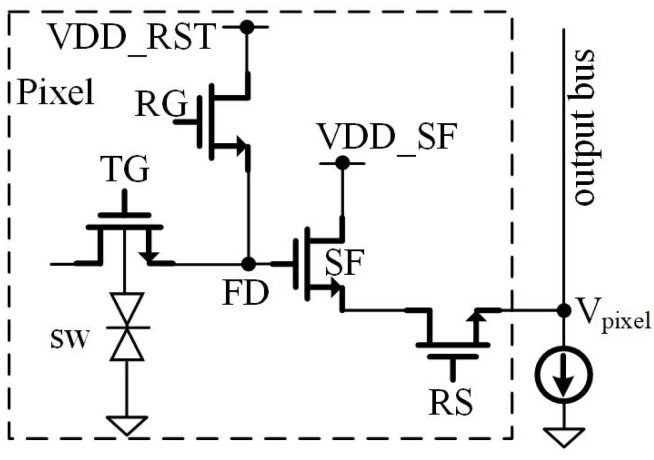
Diagram of the pump-gate jot.

This work focuses on the design of readout circuitry; therefore, the jot design is not explained in detail here. Pump-gate jots with high conversion gain and fast charge transfer speed are used in the sensor as shown in Figure 2, similar to the jot in [5].

**Figure 3:**
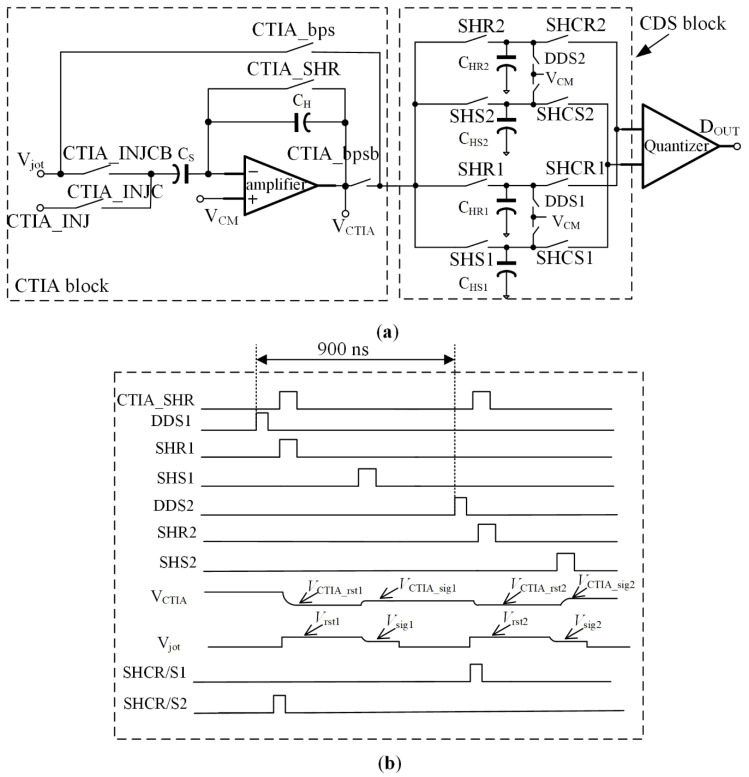
Readout chain: (**a**) diagram of the readout architecture from jot output to quantizer output and (**b**) timing diagram.

A diagram of the readout chain shown in Figure 3a consists of, from left to right, CTIA, CDS block and 1-bit quantizer. The CTIA is added before the 1-bit quantizer to place the common-mode voltage of the input signals to the quantizer in an acceptable range. In Figure 3a, V_jot_ is the jot output voltage. Two switches controlled by CTIA_bps and CTIA_bpsb are used to bypass the CTIA if needed. Two switches, controlled by a pair of signals, CTIA_INJCB and CTIA_INJC, turn off the path of V_jot_ and provide a pathway for injecting known signals to the CTIA for characterization purposes. CTIA_SHR is used for resetting the CTIA.

Figure 3b shows the accompanying timing diagram of the readout circuitry operating in normal mode. The bypass and injection functions are both disabled. The switch CTIA_bps is off, the switch CTIA_INJECB is on, and the switch CTIA_INJEC is off. There are two pairs of holding capacitors in the CDS block—C_HR1_, C_HS1_ and C_HR2_, C_HS2_—instead of only one pair of capacitors. The two pairs of capacitors work alternately, which leads to a pipeline operation of the whole readout chain. This helps save the CTIA’s power consumption, although more capacitors consume more layout area. The switch DDS1 is turned on first to clean the two readout paths of C_HR1_ and C_HS1_. Then, SHCR2 and SHCS2 are turned on so the previous values stored in holding capacitors, C_HR2_ and C_HS2_, are fed to the 1-bit quantizer and are converted to a digital code, Dout. During the quantizer conversion, the CTIA also operates. Switches CTIA_SHR and SHR1 are both turned on first. The CTIA reset voltage, V_CTIA__rst1, is sampled onto C_HS1_ before the sampling capacitor, C_S_, samples the jot reset voltage, Vrst1. After the reset, CTIA_SHR and SHR1 are both turned off. When jots output the exposure signal, Vsig1, the switch SHS1 is turned on and the C_S_ samples the jot exposure signal, Vrst1. Next, the difference of the two levels, ΔV = Vrst1 − Vsig1, is amplified by the CTIA, and the difference multiplied by the gain is V_CTIA_. The gain is determined by the ratio of C_S_ and C_H_, set to be 10. C_HS1_ samples the CTIA output, which is V_CTIA__sig1 = V_CTIA__rst1 + ΔV × C_S_/C_H_. In the meantime, a CDS operation is realized. After this, the quantizer finishes the conversion, and the CTIA finishes the amplification. The conversion result is stored in a register. The jot output is accessed during the operation of the CTIA. The conversion time of the CTIA and the quantizer are both 900 ns. A readout cluster needs to convert 2048 jot outputs into binary data per frame. The power supplies of the CTIA and the 1-bit quantizer are both 1.2 V to reduce power consumption.

The same operation will be carried out again to sample CTIA outputs to C_HR2_ and C_HS2_ while the SHCR2 and SHCS2 are turned on to feed voltages sampled on C_HR1_ and C_HS1_ to the quantizer. Therefore, the CTIA and quantizer work simultaneously to realize the pipeline operation, doubling speed for the same power consumption. However, one drawback is that two extra holding capacitors are needed to hold the previous signals, which increases the layout area. The capacitors used in the sensor are MOS capacitors with a relatively higher capacitive density than MIM capacitors. Thus, the layout area is only slightly increased.

### 3.1. CTIA

**Figure 4:**
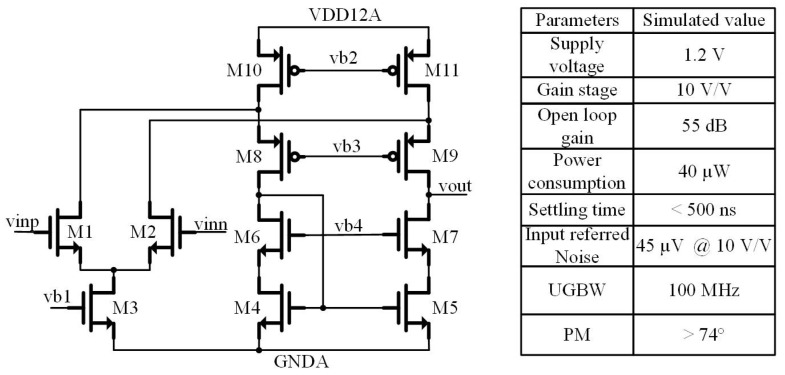
Schematic of the amplifier used in the CTIA.

The CTIA in the sensor is the key to improving the threshold uniformity of the readout clusters. A folded-cascode amplifier is chosen as the CTIA’s amplifier in consideration of the 1.2 V power supply. The schematic of the amplifier is shown in Figure 4. This sensor’s high frame rate requires the amplifier to operate at a high unity-gain bandwidth (UGBW) of 100 MHz, to realize a settling time less than 500 ns. This specification leads to high power consumption. While the pipeline operation scheme is applied to reduce power consumption, the amplifier still consumes 40 μW.

Besides the speed, the noise is another significant parameter for the sensor. The gain of the CTIA is set to be 10 to largely decrease the noise contributed by the CDS block and 1-bit quantizer. The input-referred noise PSD (power spectral density) of the amplifier is expressed as [11]:

(1) $$\begin{array}{l} \bar{S_{\mathrm{noise},\mathrm{in}}^{}}=(8kT\frac{\gamma }{g_{m1,2}})\left(1+\frac{g_{m10,11}}{g_{m1,2}}+\frac{g_{m4,5}}{g_{m1,2}}\right)+\\ \left(\frac{2K_{N}}{C_{\mathrm{ox}}f}\left(\frac{1}{{\left(WL\right)}_{1,2}}+\frac{1}{{\left(WL\right)}_{4,5}}\frac{g_{m4,5}^{2}}{g_{m1,2}^{2}}\right)+\frac{2K_{P}}{C_{\mathrm{ox}}f}\frac{1}{{\left(WL\right)}_{4,5}}\frac{g_{m10,11}^{2}}{g_{m1,2}^{2}}\right) \end{array}$$

where, *γ*, *K*_N_, *K*_P_ are parameters decided by the process and *g*_m_ is the transconductance of a transistor. Thus, to effectively reduce the noise, *g*_m1,2_ needs to be large and *g*_m10,11_ and *g*_m4,5_ need to be small.

The 1/f noise is filtered by the CDS and the *g*_m1,2_ is much larger than *g*_m4,5,10,11_. The output-referred noise power is mainly given by:

(2) $$\bar{v_{\mathrm{noise},\mathrm{out},\mathrm{amp}}^{2}}=\int_{0}^{+\infty }(8kT\frac{\gamma }{g_{m1,2}^{}}){\left|H_{\mathrm{amp}}(f)\right|}^{2}df$$

where *H*_amp_(*f*) is the noise transfer function of the CTIA during the amplification phase. With the help of the time constant of a CTIA circuit given in [11], *H*_amp_(*f*) is expressed as:

(3) $$H_{\mathrm{amp}}(f)=-\frac{C_{S}+C_{H}}{C_{H}}\frac{1}{1+s\frac{C_{L}C_{S}+C_{L}C_{H}+C_{S}C_{H}}{g_{m1,2}C_{H}}}$$

where *C_L_* is the capacitance of CHR1,2 and CHS1,2. The output-referred noise power during the amplification phase can be simplified to:

(4) $$\bar{v_{\mathrm{noise},\mathrm{out},\mathrm{amp}}^{2}}=2\gamma {\left(\frac{C_{S}+C_{H}}{C_{H}}\right)}^{2}kT\frac{C_{H}}{C_{L}C_{S}+C_{L}C_{H}+C_{S}C_{H}}$$

Then, the input-referred noise power during the amplification phase can be presented as:

(5) $$\bar{v_{\mathrm{noise},\mathrm{in},\mathrm{amp}}^{2}}=2\gamma \frac{C_{H}}{C_{S}}(1+\frac{C_{H}}{C_{S}})\frac{kT}{C_{L}+C_{H}∥C_{S}}$$

Repeat the calculation for the reset phase. The input-referred noise power during the reset phase is:

(6) $$\bar{v_{\mathrm{noise},\mathrm{in},\mathrm{rst}}^{2}}=2\gamma {\left(\frac{C_{H}}{C_{S}}\right)}^{2}\frac{4kT}{C_{L}+C_{S}}$$

Thus, the total input-referred noise power contributed by the CTIA is:

(7) $$\bar{v_{\mathrm{noise},\mathrm{in}}^{2}}=2\gamma \left({\left(\frac{C_{H}}{C_{S}}\right)}^{2}\frac{kT}{C_{L}+C_{S}}+\frac{C_{H}}{C_{S}}(1+\frac{C_{H}}{C_{S}})\frac{kT}{C_{L}+C_{H}∥C_{S}}\right)$$

Equation (7) shows that the larger the gain of the CTIA and the load capacitor and sampling capacitor, the smaller the input-referred noise.

### 3.2. CDS Block

The CDS block is used to store the CTIA reset value and the reset value plus the amplified difference between the jot reset signal and jot exposure signal. This helps remove the offset voltage contributed by the CTIA and filter out 1/f noise while the thermal noise power is doubled compared to single holding capacitor architecture. These two signals are fed to the 1-bit quantizer to determine whether there is one or more photons hitting the jot according to the threshold of the 1-bit quantizer. The values of these four holding capacitors are the same and are significant because they will largely affect the read noise and the sensor’s performance. The capacitor value also needs to be large enough to counter the kickback noise from the 1-bit quantizer. Due to the gain of the CTIA, the input referred noise is divided by 10. As a result, the capacitance is set to be 300 μF in our design.

### 3.3. 1-Bit Quantizer

**Figure 5:**
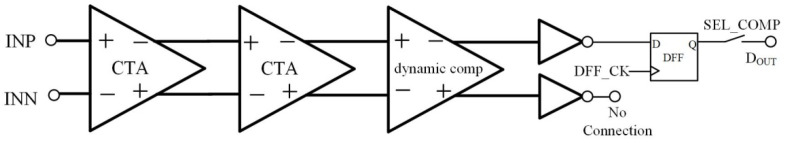
Schematic of the 1-bit quantizer.

Figure 5 shows the diagram of the 1-bit quantizer, composed of two CTAs, a dynamic comparator, two inverters, a D flip-flop (DFF) and a switch. INP and INN are the two inputs to the 1-bit quantizer from the CDS block. A two-stage cascode CTA operates as the preamplifier of the dynamic comparator. This 1-bit quantizer determines whether there is at least one photon hitting the jot or not by comparing INP and INN. Ideally, the threshold voltage of the 1-bit quantizer is 0 V. However, due to the offset contributed by the CTAs and comparator, it is slightly deviated from zero and needs to be designed as small as possible to reduce the probability of false conversion [12].

**Figure 6:**
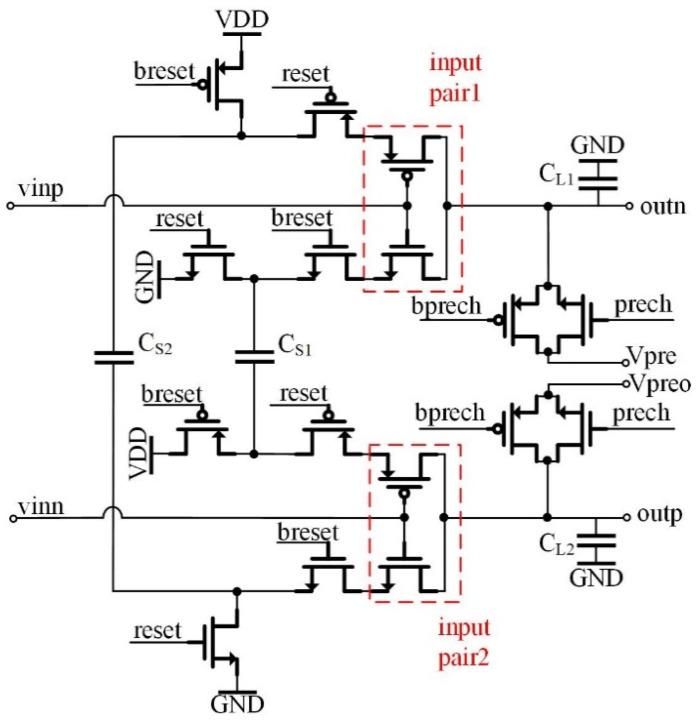
Schematic of the CTA.

Figure 6 shows the schematic of the CTA, which is designed based on the previous design [7], except for a lower power supply. Vpre and Vpreo are two reference voltages. The gain of the CTA is mainly determined by the ratio of the sampling capacitor, C_S_, and load capacitor, C_L_, which mostly comes from the gate capacitor of the next-stage’s input transistors. To adjust the threshold of the quantizer, Vpre and Vpreo of the second CTA are set to be different. Vpre and Vpreo of the first stage CTA are fixed to be the same in the sensor. This not only adjusts the threshold of the quantizer but also helps overcome the offset contributed by the dynamic comparator as well as the CTA. The random offsets of the 1-bit quantizers in different readout clusters still need to be minimized. This is because they also degrade the uniformity of the whole readout circuits.

The threshold of the quantizer can be set by adjusting Vpre and Vpreo. Each dynamic comparator’s output is connected to an inverter to match the loads of the dynamic comparator’s two output nodes, although one output is unused. This helps reduce the offset of the dynamic comparator. The output of one inverter drives the DFF, which works as a register to store the conversion result of the 1-bit quantizer, and it is readout during the next quantizer conversion.

### 3.4. Digital Blocks

In this sensor, since the readout is designed in cluster-parallel architecture instead of column-parallel style, a large clock tree is used for driving the entirety of the readout circuitry. The advantages of utilizing the clock tree are to balance clock paths and lower timing skew [13].

**Figure 7:**
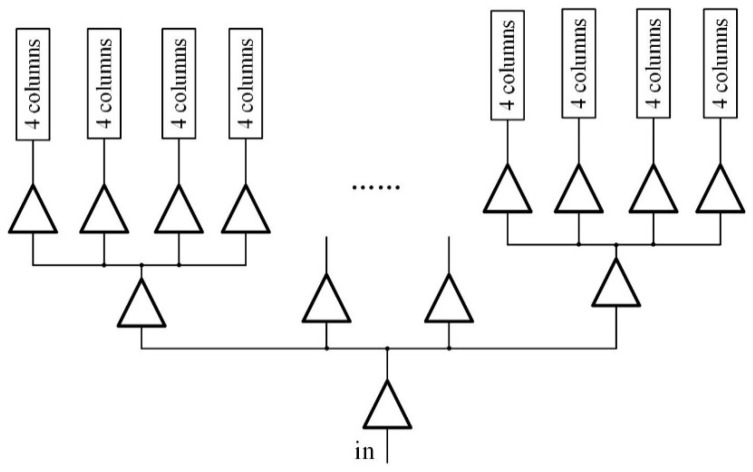
Distribution of the clock tree.

Figure 7 shows the distribution of the clock tree. Each buffer at the end point drives four columns, or 64 (16 × 4) readout clusters in total. The propagation delay caused by this architecture is 100 times smaller than the simple clock buffer driving all columns, which usually needs to drive more than 1000 columns. In addition, it is more difficult to synchronize the timing signals for the first and last columns for simple clock driver than for clock tree driver.

The large clock tree also has some drawbacks. Many digital buffers are required and are located very close to the array to drive it, which could easily cause large spikes in the digital power supply. Some schemes are utilized in the chip design to ensure this will not degrade the performance of the readout circuitry. The whole clock tree is laid in a deep nwell so these dirty digital signals are isolated from analog signals. As many decoupling capacitors are put near the clock tree block as possible to provide enough local charge for the rising/falling of timing signals. The coupling between digital signals and sensitive analog signals are avoided as well. The width of the routes of digital power supplies should be large enough to mitigate the voltage drop caused by metal resistance.

CMOS buffers are used to send digital data outside the chip instead of low-voltage differential signaling (LVDS) transmitters. This consumes many pads to guarantee the data transfer rate. Thus, the pad numbers used for analog power supplies are largely limited and the performance of the readout clusters might be degraded. In future designs, LVDS transmitters will be implemented in the sensor.

## 4. Common-Mode Voltage Variation Improvement

As mentioned previously, the 1-bit quantizer in the previous sensor had significant threshold non-uniformity, probably due to the fluctuation of the common-mode voltage of the jot output. The common-mode voltage variation causes the CTA gain to change, leading to threshold variation. This is because the quantizer threshold is (Vpre − Vpreo)/gain. A complete readout-circuit redesign was performed to address this issue. A CTIA [14] is added before the 1-bit quantizer to guarantee the common-mode voltage of input signals to the quantizer is in an acceptable range. However, it is also important to make sure that the added CTIA does not introduce more offset variation. Otherwise, the thresholds of different readout clusters will still vary dramatically.

**Figure 8:**
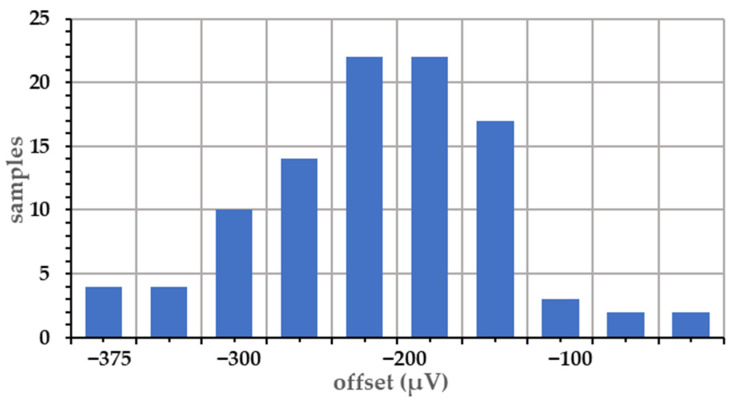
Amplifier offset distribution from 100 Monte-Carlo simulation runs.

Figure 8 shows the output offset voltage of the amplifier in the CTIA based on 100 post-layout Monte-Carlo runs. The simulation results follow a normal distribution. The offset range is 0 to −400 μV and the input-referred offset voltage is from 0 to −40 μV, taking into account the 10× gain of the CTIA. The mean of the offset voltage is not zero due to the clock feedthrough and charge sharing of switches in the CTIA. The input-referred offset voltage is relatively small compared with an estimated jot 340 μV/e− CG and the CDS block removes the offset voltage from the CTIA. Thus, the CTIA design is highly consistent. Furthermore, the ±22 μV offset contributed by the quantizer divided by the CTIA 10× gain is also low, which causes little effect on the uniformity.

Without the CTIA, the common-mode voltage variation is mainly caused by the threshold variation of the SF in the jot. It is more than 50 mV. With the CTIA, the variation is the output offset of the CTIA plus the offset from the quantizer, which is less than −400 μV according to the results above. Thus, the common-mode voltage fluctuation is largely improved.

## 5. Measurement Results

**Figure 9:**
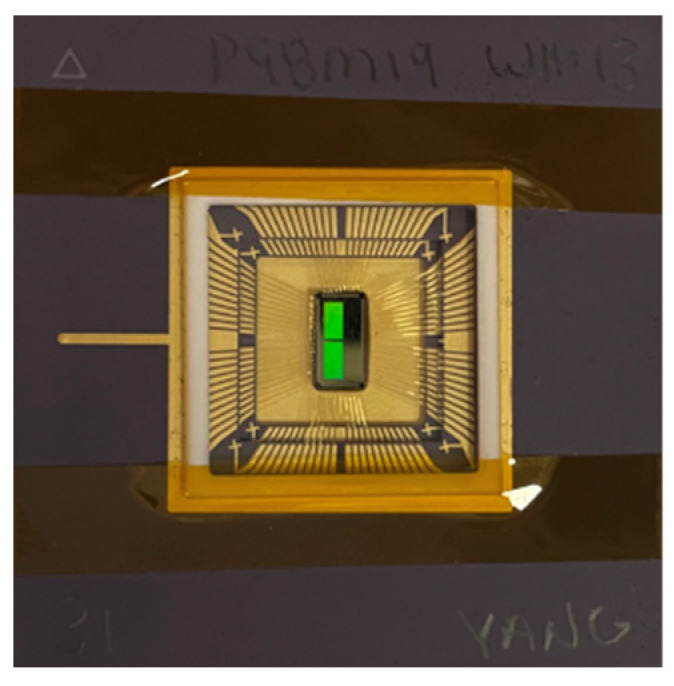
QIS chip.

The single-bit QIS was fabricated in a TSMC 45 nm/65 nm stacked BSI CIS process and the chip area is 6 mm × 3 mm. A photo of the packaged die is shown in Figure 9.

The main purpose of this work was to redesign the readout circuitry and improve the threshold uniformity. Thus, mainly the readout circuity testing results are reported here.

**Figure 10:**
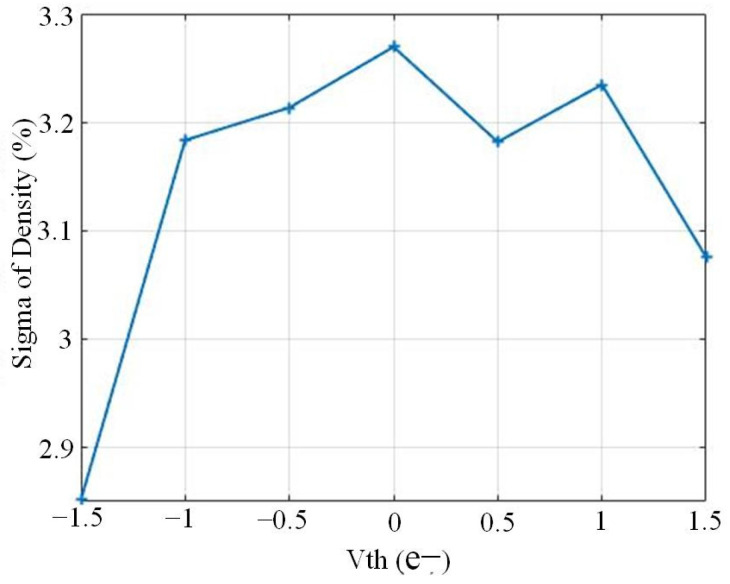
Variation of readout chain using statistical approach from measurements.

The readout chain variation was verified at full speed, 500 fps, with the jot reset gates always on, keeping the input of the readout chain constant. The output density of the quantizer was measured by sweeping the threshold voltage over the equivalent range from −1.5 e− to +1.5 e−, calibrated from simulation results. Twenty readout clusters were measured, each with 10k samples of the same jot within that cluster. A bit density (due to readout noise) was determined for each cluster, and the standard deviation of the 20 cluster bit densities for each threshold is presented in Figure 10 as a function of threshold voltage. The standard deviation of the readout chain is about 3.2% in the central region for this statistical measurement.

**Figure 11:**
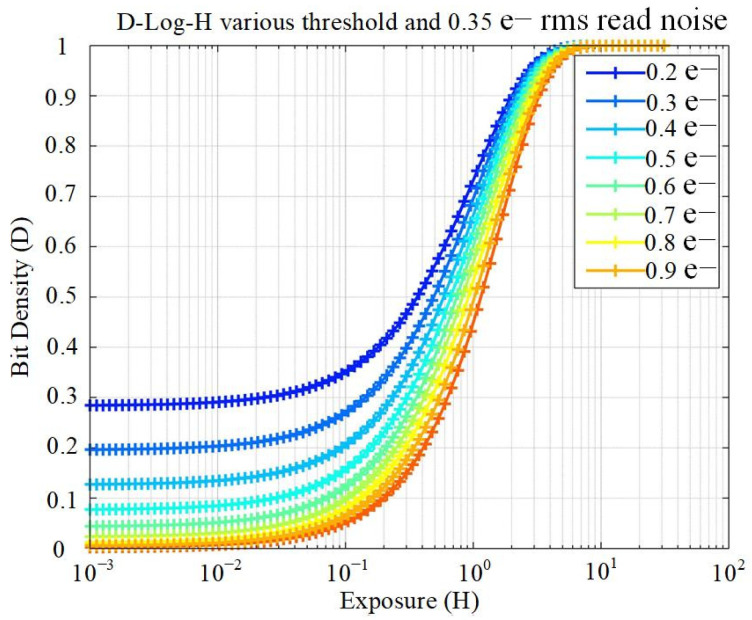
Theoretical bit density vs. exposure for different thresholds (input-electron referred) with 0.35 e− rms read noise [15].

The D-log-H characteristic of a single-bit QIS is a useful tool for evaluating uniformity as well as proving true single-electron sensitivity. Figure 11 shows the theoretical D-log-H for a single-bit QIS with 0.35 e− rms read noise and various quantizer threshold levels [15]. The distribution becomes narrower and bit density smaller at lower exposures with higher threshold. If the threshold uniformity across all readout clusters is good, the spread of all clusters’ D-log-H curves is narrow. For the D-log-H characteristics of a readout cluster with a fixed quantizer threshold, the read noise would cause an increase to the bit density in low light levels.

**Figure 12:**
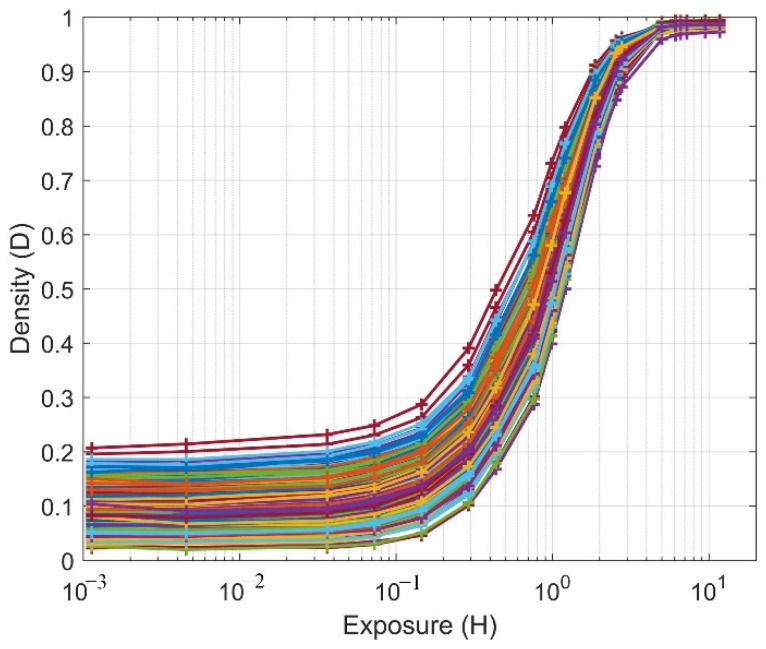
Measured D-log-H for all clusters, demonstrating good uniformity of threshold from 0.3 to 0.8 e−.

**Figure 13:**
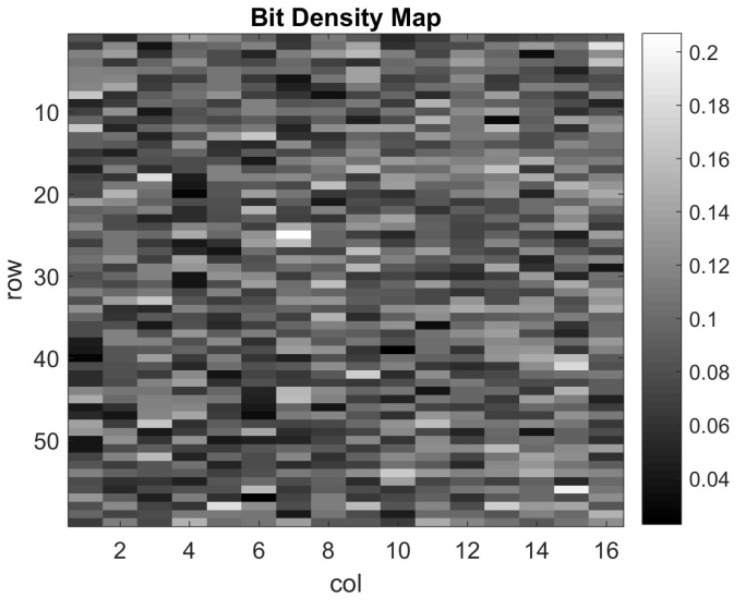
Bit density map for all clusters at H = 10^−3^.

**Figure 14:**
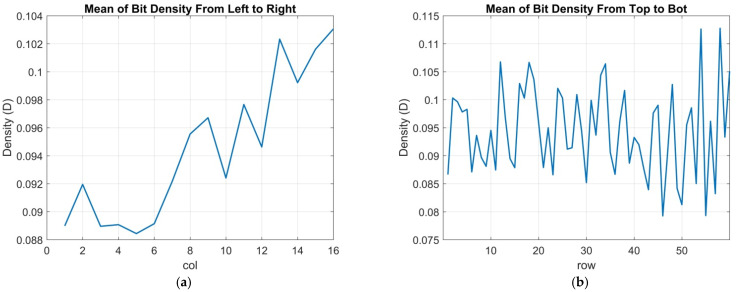
Average bit densities: (**a**) column average and (**b**) row average.

The threshold uniformity of the whole array, D-log-H curves from the 2Mjot sensor for all 1024 readout clusters, were measured and are depicted in Figure 12. To estimate the thresholds of the different clusters, Figure 12 is compared with the theoretical D-log-H curves with 0.35 e− read noise, and the range of all clusters’ thresholds is from 0.3 to 0.8 e−. To show the variation of all clusters more clearly, Figure 13 shows a map of all cluster bit densities at the H = 10^−3^ of the D-log-H curves. From the figure, no clear trend can be found, meaning that the variation for all clusters is uniform. Then, to analyze the uniformity quantitatively, the bit density average of each column and row are both calculated as shown in Figure 14. A trend can be seen for column average in Figure 14a while still no trend is shown for row average. There are some possible reasons: the nonuniform distribution of oxide thickness, the timing delay and the IR drop across the whole array. The most probable one is the distribution of three CTIA power supply pads, located at the top, left and bottom of the cluster’s array.

**Figure 15:**
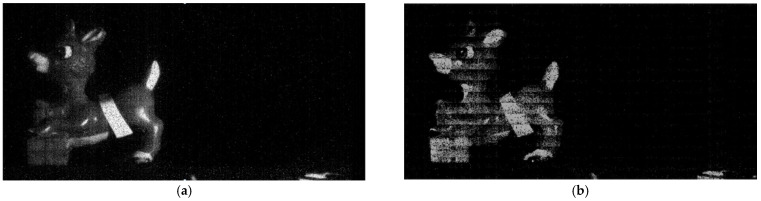
Sample image acquired with 0.5 e− threshold, (**a**) 230 fps and (**b**) 500 fps.

Sample images, shown in Figure 15, were also acquired at different speeds to demonstrate the performance of the imager. The readout circuit threshold was set to be 0.5 e−, and 500 frames were taken and summed. The offset and noise contributed by the readout circuit were subtracted from the images shown, which were acquired under dark conditions. When frame rate is 230 fps, the reindeer image is clear. However, when the frame rate is increased to 500 fps, the image quality becomes worse and row fixed-pattern noise (FPN) can be seen. However, this is not what we expected. A possible reason can be row timing skew.

## 6. Conclusions

A 2048 × 1024 high-speed single-bit QIS test chip for improving readout cluster uniformity was implemented with a 45 nm/65 nm stacked BSI CIS process and was tested. The 1-bit quantizer based on CTA readout circuits in the previous single-bit QIS had significant threshold non-uniformity due to the fluctuation of the common-mode voltage of the jot output. The change of common-mode voltage causes the change in CTA gain, leading to threshold variation. To improve the performance of the 1-bit quantizer, a CTIA is added before a 1-bit quantizer to guarantee the stability of the common-mode voltage of the input signals fed to the 1-bit quantizer compared with the previous design. A pipeline operation mode is also proposed so that the CTIA and the 1-bit CTIA can work at the same time to reduce the CTIA’s power consumption. A good threshold uniformity in the range of 0.3 to 0.8 e− extracted from measured D-log-H results for all readout clusters is demonstrated at 500 fps equivalent timing. However, the image taken at 500 fps shows FPN, while a clean image can be captured with a lower speed, 230 fps. Thus, the speed of the single-bit QIS still needs to be further increased in the future.

## Data Availability

Data are contained within the article.
